# A Genome-Wide Identification and Comparative Analysis of the Heavy-Metal-Associated Gene Family in Cucurbitaceae Species and Their Role in *Cucurbita pepo* under Arsenic Stress

**DOI:** 10.3390/genes14101877

**Published:** 2023-09-27

**Authors:** Gerardo Flores-Iga, Carlos Lopez-Ortiz, Celeste Gracia-Rodriguez, Aldo Almeida, Padma Nimmakayala, Umesh K. Reddy, Nagamani Balagurusamy

**Affiliations:** 1 Laboratorio de Biorremediación, Facultad de Ciencias Biológicas, Universidad Autónoma de Coahuila, Torreón 27275, Coahuila, México; juan.iga@wvstateu.edu (G.F.-I.); celeste.rodreguez@wvstateu.edu (C.G.-R.); 2 Gus R. Douglass Institute, Department of Biology, West Virginia State University, Institute, WV 25112-1000, USA; carlos.ortiz@wvstateu.edu (C.L.-O.); padma@wvstateu.edu (P.N.); 3 Department of Plant and Environmental Sciences, University of Copenhagen, 1871 Frederiksberg, Denmark; robles@plen.ku.dk

**Keywords:** HMA, P1B-type ATPase, arsenic stress, arsenic transport, Cucurbits

## Abstract

The heavy-metal-associated (HMA) proteins are a class of P_B1_-type ATPases related to the intracellular transport and detoxification of metals. However, due to a lack of information regarding the HMA gene family in the Cucurbitaceae family, a comprehensive genome-wide analysis of the HMA family was performed in ten Cucurbitaceae species: *Citrullus amarus*, *Citrullus colocynthis*, *Citrullus lanatus*, *Citrullus mucosospermus*, *Cucumis melo*, *Cucumis sativus*, *Cucurbita maxima*, *Cucurbita moschata*, *Cucurbita pepo*, and *Legenaria siceraria*. We identified 103 Cucurbit HMA proteins with various members, ranging from 8 (*Legenaria siceraria*) to 14 (*Cucurbita pepo*) across species. The phylogenetic and structural analysis confirmed that the Cucurbitaceae HMA protein family could be further classified into two major clades: Zn/Co/Cd/Pb and Cu/Ag. The GO-annotation-based subcellular localization analysis predicted that all HMA gene family members were localized on membranes. Moreover, the analysis of conserved motifs and gene structure (intron/exon) revealed the functional divergence between clades. The interspecies microsynteny analysis demonstrated that maximum orthologous genes were found between species of the *Citrullus* genera. Finally, nine candidate HMA genes were selected, and their expression analysis was carried out via qRT-PCR in root, leaf, flower, and fruit tissues of *C. pepo* under arsenic stress. The expression pattern of the CpeHMA genes showed a distinct pattern of expression in root and shoot tissues, with a remarkable expression of *CpeHMA6* and *CpeHMA3* genes from the Cu/Ag clade. Overall, this study provides insights into the functional analysis of the HMA gene family in Cucurbitaceae species and lays down the basic knowledge to explore the role and mechanism of the HMA gene family to cope with arsenic stress conditions.

## 1. Introduction

Cucurbits are one of the major crop families with high economic value and are widely cultivated worldwide. Four genera, *Cucurbita* (squash and pumpkins), *Cucumis* (cucumbers and melons), *Citrullus* (watermelons), and *Lagenaria* (bottle gourd), are among the ten most economically significant vegetable crops in the world, while numerous others have regional significance [1]. Nevertheless, the global Cucurbit growth and yield are adversely influenced by environmental stresses such as droughts, salinity, and high concentrations of heavy metals and metalloids [2,3].

Arsenic (As) is a non-essential metalloid ubiquitous in soil at low levels; however, agricultural soils are threatened by toxic contamination from anthropogenic activities, leading to excessive accumulation of arsenic [4]. Its presence in polluted environmental conditions such as groundwater and cropping soil causes severe threats to living organisms, including plants and, consequently, humans [5,6].

Plants have developed various adaptation strategies to protect themselves from harmful environmental conditions, including accumulating and transporting heavy metals and metalloids [7]. Membrane transport plays a vital role in heavy metal detoxification, allowing absorption and transport of many cations from the root to the shoot and redistribution among aerial parts [8,9]. Among the different membrane transporters, the P_1B_-type ATPase, also known as the heavy metal ATPase (HMA), which belongs to the large P-type ATPase family, plays an important role in heavy metal transport [10,11,12].

HMAs transport essential metal ions required for plant growth and development, such as Cu^2+^ and Zn^2+^, and distribute non-essential heavy metal ions, including Cd^2+^, Co^2+^, and Pb^2+^. Typical HMA proteins contain the E1–E2 ATPase domain and a haloacid dehalogenase-like hydrolase domain. Additionally, both sides of the N-terminal and C-terminal metal-binding sites may possess one or more soluble metal-binding domains (MBDs) that interact with or bind to specific metal ions [8,13,14]. The HMA domain is also located in P_1B_-type ATPases, a heavy-metal-associated regulatory domain [15,16]. Based on metal substrate specificity, HMAs can be clustered into two major phylogenetic subclasses, namely, the Cu/Ag P_1B_-ATPase group and the Zn/Co/Cd/Pb P_1B_-ATPase group [17].

HMA genes have been identified in the model and non-model plants with a different number of genes and diversification patterns, including *Arabidopsis thaliana* [15], *Oryza sativa* [18], *Populus trichocarpa* [19], *Glycine max* [20], *Zea mays* [21], *Sorghum bicolor* [21], *Hordeum vulgare* [22,23], *Linum usitatissimum* [24], *Brassica napus* [25], *Pyrus bretschneideri* [26], *Morus alba* [27], *Medicago truncatula* [28], and *Fagopyrum tataricum* [29]. The functions of HMA genes have been comprehensively studied; for instance, in *A. thaliana*, *AtHMA1* is involved in exporting Zn from the chloroplast [30], while the overexpression of *AtHMA3* enhances tolerance and accumulation of Cd, Zn, Pb, and Co in plants [31]. Likewise, overexpression of *SpHMA3* in *Sedum plumbizinccicola* has been reported to confer Cd hyper-tolerance [32]. Moreover, it has been shown that *OsHMA5* is involved in the shoot translocation of Cu^2+^ in rice [33], whereas *OsHMA1* and *OsHMA3* are involved in the Zn and Cd transport, respectively [18]. Furthermore, eight HMA proteins have been identified in cucumber as a response to cadmium accumulation. Although these proteins have not been further characterized, *CsHMA3* and *CsHMA4* have been associated with transporting cadmium, lead, and zinc from the root to the stem [34].

Although, HMA genes play a vital role in heavy metal transmembrane trafficking in different plants [16]. To date, a single study has reported the function of P-type ATPase involved in active arsenic transport in *Pteris vitatta* [35]. Nevertheless, the identification and functional characterization of the HMA gene family in Cucurbit species and their expression under As stress have not been previously evaluated. Thus, in this study, we performed a systematic genome-wide identification and comparative analysis of the HMA family in *Citrullus amarus*, *Citrullus colocynthis*, *Citrullus lanatus*, *Citrullus mucusospermus*, *Cucumis melo*, *Cucumis sativus*, *Cucurbita maxima*, *Cucurbita moschata*, *Cucurbita pepo*, and *Legenaria siceraria*. HMA genes were identified and studied in terms of their chromosomal location and synteny, phylogeny, conserved motifs, structure, and expression profiles in different tissues of *C. pepo* under copper treatments, a recognized heavy metal related to the HMA family, as well as their expression under arsenic stress conditions. Therefore, our results provide insights for future investigations into the roles of HMA genes in Cucurbitaceous plants and other species.

## 2. Materials and Methods

### 2.1. Identification of HMA Genes in Cucurbits

For the identification of members of the HMA gene family in Cucurbit species (*C. amarus*, *C. colocynthis*, *C. lanatus*, *C. mucusospermus*, *C. melo*, *C. sativus*, *C. maxima*, *C. moschata*, *C. pepo*, and *L. siceraria*), the Basic Local Alignment Search Tool (BLAST) of the Cucurbit genomics database v2 (CuGenDB; http://cucurbitgenomics.org/v2, accessed on 15 October 2022) was employed using all the Arabidopsis HMA genes as queries [15,36]. To ensure the presence of conserved domains E1-E2 ATPase (IPR008250), hydrolase (IPR041492), and HMA (IPR006121) in the identified HMA proteins, the Pfam database [37] and the NCBI Conserved Domain Database (https://www.ncbi.nlm.nih.gov/Structure/cdd/wrpsb.cgi, accessed on 15 October 2022) were used. Sequences with no HMA-related domains were excluded from a further analysis. Moreover, genomic, coding sequence (CDS), and promoter region sequences were downloaded from the CuGenDB for confirmed genes. Further, physiochemical parameters such as molecular weight, polypeptide length, and theoretical isoelectric point (pI) value were calculated using ExPASy ProtParam software (http://web.expasy.org/protparam/, accessed on 15 October 2022). The identified HMA genes were *named CamHMA1-CamHMA10*; *CcoHMA1-CcoHMA9*; *ClaHMA1-ClaHMA10*; *CmuHMA1-CmuHMA9*; *CmeHMA1-CmeHMA10*; *CsaHMA1-CsaHMA9*; *CmaHMA1-CmaHMA12*; *CmoHMA1-CmoHMA12*; *CpeHMA1-CpeHMA14*; and *LsiHMA1-LsiHMA8* using the prefix “Cam”, “Cco”, “Cla”, “Cmu”, “Cme”, “Csa”, “Cma”, “Cmo”, “Cpe”, and “Lsi” for *C. amarus*, *C. colocynthis*, *C. lanatus*, *C. mucusospermus*, *C. melo*, *C. sativus*, *C. maxima*, *C. moschata*, *C. pepo*, and *L. siceraria*, respectively, followed by “HMA” for the heavy-metal-associated domain and lastly, the progressive number according to their chromosome number and chromosomal positions. *A. thaliana* HMA sequences were downloaded from the Arabidopsis Information Resource (TAIR, http://www.arabidopsis.org/index.jsp, accessed on 15 October 2022, release 10.0).

### 2.2. Chromosomal Location and Gene Structure of HMA Proteins

Physical chromosome location data for each HMA gene in the Cucurbit species were obtained from the CuGenDB database and then displayed by using the MapGene2Chromosome V2 (http://mg2c.iask.in/mg2c_v2.0/, accessed on 15 October 2022) in each chromosome where an HMA gene was found. Moreover, an exon–intron gene structure analysis was carried out by submitting merged General Feature Format (GFF3) files of Cucurbitaceous plants to the Biosequence structure tool in TBtools [38].

### 2.3. Phylogenetic Analysis, Synteny Analysis, and Gene Duplication Events of the Cucurbit HMA Family

The amino acid sequences of the Cucurbit species and Arabidopsis were imported into MEGA 7 [39], and multiple sequence alignment was performed using ClustalW [40] with gap-open and gap-extension penalties of 10 and 0.1, respectively. Alignment was used to build a phylogenetic tree based on the neighbor-joining (NJ) method. After a bootstrap analysis with 1000 replicates, the tree was exported into Newick format to display it by using iTOL software (version 6.8.1, http://itol.embl.de/index.shtml, accessed on 15 October 2022).

A syntenic relationship analysis of the HMA gene family between species of *Citrullus*, *Cucumis*, and *Cucurbita* genera was carried out and visualized using TBTools with the E-value < 1× 10^−10^ [38]. Non-synonymous (Ka), synonymous substitution (Ks), and Ka/Ks ratios for duplicated gene pairs were calculated in the Ka/Ks calculation tool (http://services.cbu.uib.no/tools/kaks, accessed on 15 May 2023) using the CDS of Cucurbits. The duplication date was estimated according to the following formula, Million Years Ago (MYA) = Ks/2λ, assuming a clock-like rate (λ) of 6.56 synonymous substitutions per 10^−9^ years [41].

### 2.4. Motif Analysis and Promoter Cis-Element Identification

HMA proteins were subjected to the Multiple Em for Motif Elicitation (MEME) tool found in the MEME suite (https://meme-suite.org/meme/, accessed on 15 October 2022) to identify common and species-specific motifs in the Cucurbit proteins. The analysis was performed with a maximum number of motifs of ten and an optimum motif width from 6 to 50. To identify motif function, discovered MEME motifs were searched in the ExPASy-PROSITE database using the ScanProsite tool (https://prosite.expasy.org/scanprosite/, accessed on 15 October 2022). Furthermore, promoter sequences (2000 bp upstream) from Cucurbit HMA genes were submitted to The PlantCARE database (http://bioinformatics.psb.ugent.be/webtools/plantcare/html, accessed on 15 October 2022) to analyze potential cis-regulatory elements within promoter sequences of the Cucurbit HMA genes.

### 2.5. Gene Ontology (GO) Annotation of HMA Proteins

The functional annotation, including cellular component, molecular function, and biological process of HMA proteins, was performed using OmicsBox Software (https://www.biobam.com/omicsbox/, accessed on 15 November 2022). The amino acid sequences of HMA proteins were imported into the OmicsBox program to execute three steps: (1) BLASTp against the NCBI non-redundant protein database, (2) mapping and retrieval of GO terms associated with the BLAST results, and (3) annotation of GO terms associated with each query to relate the sequences to known protein function.

### 2.6. Expression Pattern of the HMA Family in C. pepo under Different Cu Treatments

RNA-seq gene expression data of CpeHMA genes were retrieved from the NCBI GEO DataSets (Accession: GSE173716) from a previously published work by Marmiroli et al. [42]. The fragments per kilobase transcripts per million mapped reads (FPKM) expression values for root, leaf, and pollen tissues from *C. pepo* exposed to copper oxide (CuO) nanoparticles, bulk CuO (100 mg kg^−1^), and CuSO_4_ (320 mg kg^−1^) were used to generate a heatmap and compare the expression of *CpHMA*1-14 genes identified by using the tydir and ggplot2 packages (https://ggplot2.tidyverse.org/, accessed on 15 July 2023).

### 2.7. Expression Pattern with RT-qPCR of the HMA Family in Cucurbita pepo under As Treatment

*C. pepo* var. cylindrica “Golden” seeds were germinated directly in the soil. Seedlings were transferred to 0, 50, 100, and 200 µM As (V) soil treatments and irrigated with the same water concentration every other day. Roots, leaves, flowers, and fruit tissues were collected at the anthesis stage, as previously reported by Obrero et al. [43,44]. Tissues were immediately frozen in liquid nitrogen and stored at −80 °C. Further, total RNA was isolated from roots, leaves, flowers, and fruits of *C. pepo* according to the E.Z.N.A. Plant RNA Kit (Omega Bio-Tek, Norcross, GA, USA) following manufacturer instructions. An equivalent concentration of total RNA of the different tissues was used to synthesize first-strand cDNA with the SuperScript™ IV First-Strand Synthesis System (Invitrogen, Waltham, MA, USA). Equal cDNA concentration of samples was used for the qRT-PCR analysis. A StepOnePlus Real-Time PCR system (Applied Biosystems, Foster City, CA, USA) was employed for the qRT-PCR analysis with a final reaction volume of 20 µL containing 1 μL of cDNA template, 2 μL of forward and reverse primer at 10 μM, 10 μL of SYBR Green PCR Master (ROX) (Roche, Shanghai, China), and 7 μL of nuclease-free water. Three replicates per treatment were run to compute the average Ct values that were further analyzed with the 2^−ΔΔCt^ method [45]. The Actin gene was used as an endogenous control to normalize the relative gene expression. HMA primers for qRT-PCR were designed in Primer3Plus software (version 3.3.0) [46] and are listed in Table S1.

## 3. Results

### 3.1. Identification of HMA Genes in Cucurbits

A total of 103 genes potentially encoding HMA proteins were identified and classified: 10 from *C. amarus*, 9 from *C. colocynthis*, 10 from *C. lanatus*, 9 from *C. mucusospermus*, 10 from *C. melo*, 9 from *C. sativus*, 12 from *C. maxima*, 12 from *C. moschata*, 14 from *C. pepo*, and 8 from *L. siceraria* (Table 1). Moreover, amino acid length, molecular weight, and isoelectric point of Cucurbit HMA proteins were deducted from their protein sequences and are listed in Table S2. The protein length of Cucurbit HMA proteins generally varied between 356 and 1251 amino acid residues. The molecular weight was determined to range from 37.8 KDa to 143.5 KDa, while the isoelectric point (pI) ranged from 4.96 pH to 9.23 pH.

### 3.2. Chromosomal Location and Gene Structure of HMA Genes

**Figure 1:**
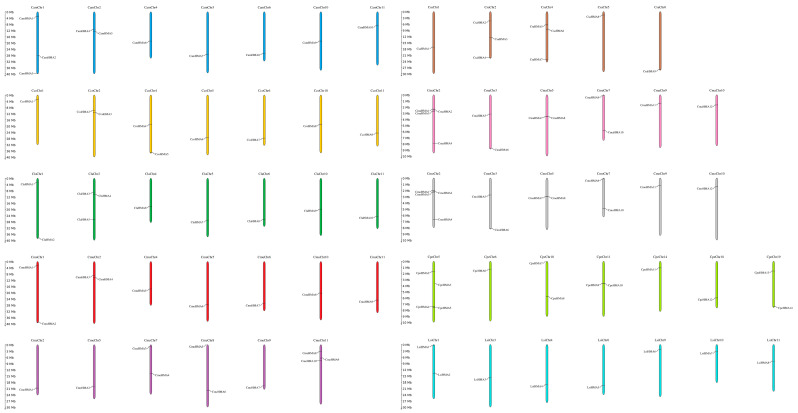
Chromosomal locations of HMA genes in *C. amarus* (dark blue), *C. colocynthis* (yellow), *C. lanatus* (dark green), *C. mucusospermus* (red), *C. melo* (purple), *C. sativus* (brown), *C. maxima* (pink), *C. moschata* (gray), *C. pepo* (light green), and *L. siceraria* (light blue). Chromosome numbers are represented at the top of each chromosome. The left panel scale indicates the chromosome length in Mb.

HMA genes in Cucurbits were found to be located in various chromosomes with a wide distribution, except for CpeHMA1 from *C. pepo* found on the unanchored scaffold. Nevertheless, not all Cucurbit species showed the presence of HMA genes in all of their chromosomes, as depicted in Figure 1. HMA genes were detected in chromosomes 1, 2, 4, 5, 6, and 10 from *Citrullus* plants; 2, 5, 7, 8, 9, and 11 of *C. melo*; 1, 2, 4, and 6 of *C. sativus*; 2, 3, 5, 7, 9, and 10 of *C. maxima* and *C. moschata*; 5, 6, 10, 11, 14, 18, and 19 of *C. pepo*; and 1, 3, 4, 6, 9, 10, and 11 of *L. siceraria.* Particularly, *C. amarus* and *L. siceraria* have the highest number of HMA proteins in Chr1 with three and two members, respectively, while *C. lanatus*, *C. maxima*, and *C. moschata* in Chr2 have three, four, and four members, respectively. Similarly, *C. melo* has the highest number of HMA proteins in Chr11 with three members, while *C. pepo* in Chr5 has 4. Furthermore, similar quantities of members in not only one chromosome of several species are present due to tandem duplicates; *C. colocynthis* and *C. sativus* have the highest amount in Chr2 and Chr4 with two and three members each, respectively, and *C. mucosospermus* has the same highest amount in Chr1 and Chr2 with two members each.

**Figure 2:**
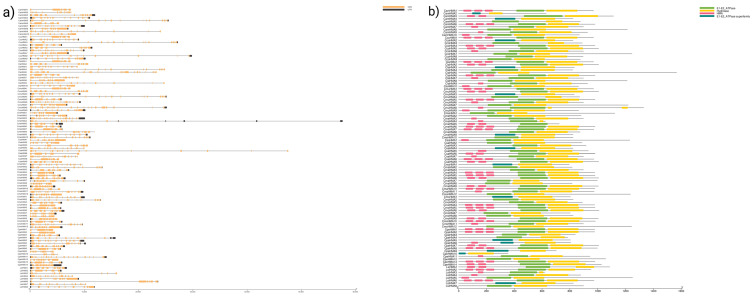
Gene structure and protein domains of the HMA family in Cucurbit species. (**a**) Exon/intron structures of HMA genes. Exons are represented as orange boxes, introns as strings, and promoter UTRs in black. (**b**) The protein domain structure pattern of HMA gene family, and different-colored rectangles represent different structural domains; the green rectangles represent E1–E2 ATPase; the yellow rectangle represents hydrolase; the pink rectangle represents HMA; the dark-green rectangle represents E1–E2 ATPase superfamily. The gene name is on the left side of each sequence, and the below scale indicates the length in kb and aa, respectively.

Next, we utilized a biosequence structure tool to produce gene structure schematic diagrams and assess the exon/intron arrangement of coding and genome sequences in HMA genes across Cucurbit species. Our analysis of the gene structures indicated significant variation in intron positions, lengths, and numbers across all species studied. Nonetheless, members most closely related shared similar exon/intron structures either according to the number of introns or exon length. Specifically, the number of exons present in *Citrullus* species ranged from 3 to 25, ranged from 3 to 17 in *Cucumis* species, ranged from 5 to 19 in *Cucurbita* species, and ranged from 6 to 16 in *L. siceraria* species. The detailed gene structure of the Cucurbit HMA genes is in Figure 2a. Further, we also observed that the location of the domains in the HMA proteins follows the forward pattern reported in Arabidopsis [36], i.e., HMA proteins in the clade of Cu/Ag showed HMA, E1-E2_ATPase, and hydrolase domains, while HMA proteins in the Zn/Co/Cd/Pb exhibited only the E1-E2_ATPase and hydrolase domains (Figure 2b).

### 3.3. Phylogenetic Analysis, Synteny Analysis, and Gene Duplication Events of the Cucurbit HMA Gene Family

**Figure 3:**
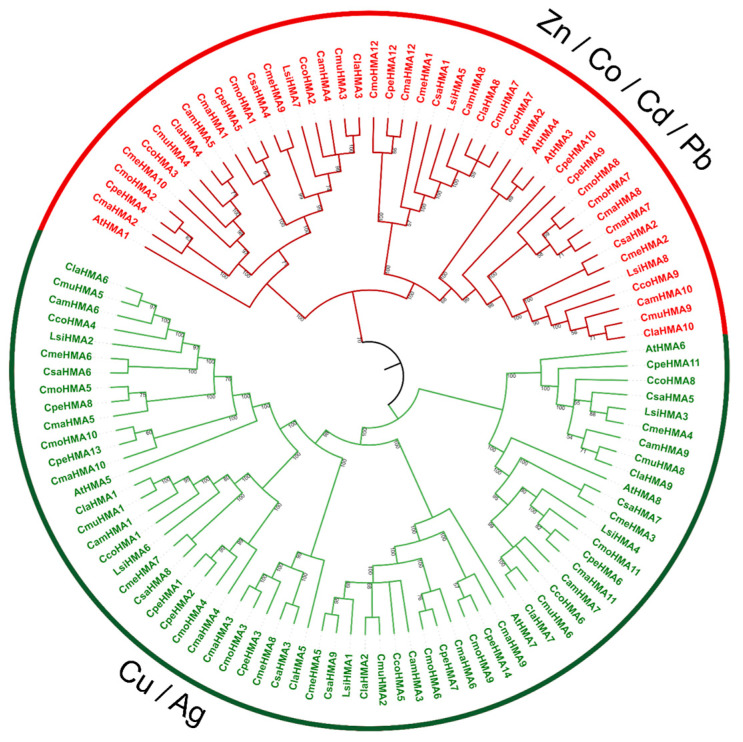
Phylogenetic relationship of the HMA gene family. Phylogenetic analysis of Cucurbits and *Arabidopsis* was carried out using the neighbor-joining method with 1000 bootstraps. Based on genetic and functional studies, the phylogeny was divided into two major clades, P1B-ATPases; zinc (Zn)/cobalt (Co)/cadmium (Cd)/lead (Pb) group is highlighted in red, and the copper (Cu)/silver (Ag) group is highlighted in green.

To examine the phylogenetic relationships among the Cucurbit HMA proteins, an unrooted phylogenetic tree was constructed from alignments of the 103 full-length HMA sequences (Figure 3). The HMA proteins were classified into two major clades, the Zn/Co/Cd/Pb and the Cu/Ag. While the number of HMA genes in *Citrullus*, *Cucumis*, and *L. siceraria* species was similar compared to *Arabidopsis* [36], *Cucurbita* species exhibited distinct, independent duplication events. Specifically, four paralog pairs (*CmaHMA7-CmaHMA8*, *CmoHMA7-CmoHMA8*, *CpHMA1-CpHMA2*, and *CpeHMA9-CpeHMA10*) were found.

**Figure 4:**
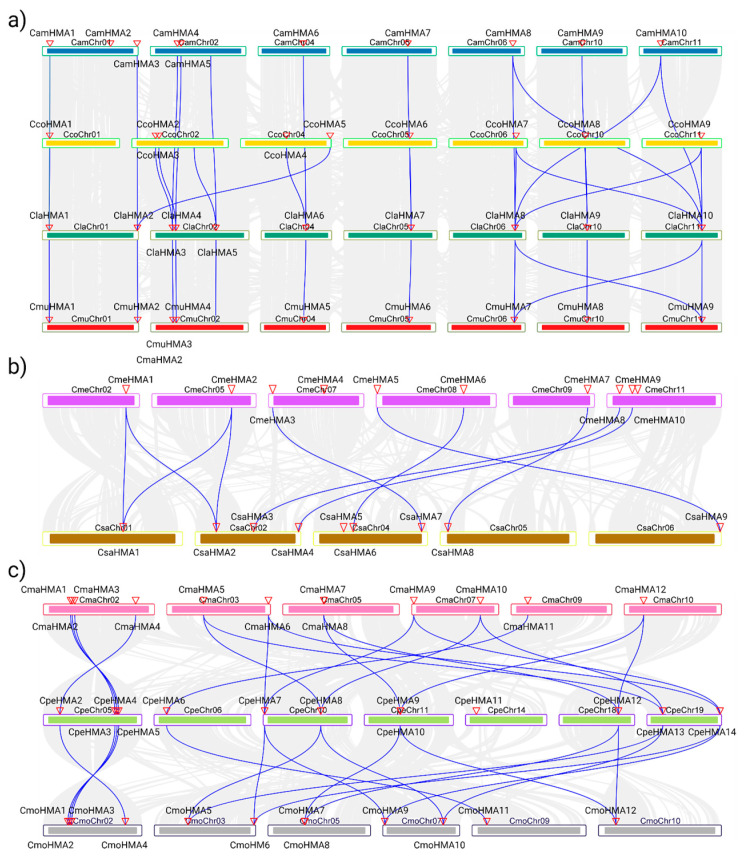
Collinearity analysis between the HMA gene family in Cucurbitaceae species. (**a**) Synteny between the *Citrullus* genera: *C. amarus* (dark blue), *C. colocynthis* (yellow), *C. lanatus* (dark green), and *C. mucusospermus* (red). (**b**) Synteny between the *Cucumis* genera: *C. melo* (purple) and *C. sativus* (brown). (**c**) Synteny between the *Cucurbita* genera: *C. maxima* (pink), *C. pepo* (light green), and *C. moschata* (gray). The collinearity analysis was visualized using TBTools with an E-value < 1 × 10^−10^.

The HMA genes from *Citrullus*, *Cucumis*, and *Cucurbita* species were subjected to synteny and a gene duplication analysis to confirm further the results from the phylogenetic tree (Figure 4). The syntenic analysis among the HMA genes of the three genera revealed collinearity among species; in the *Citrullus* genera, specifically between *C. amarus* and *C. lanatus* with ten events, while in the *Cucurbita* genera, between *C. maxima* and *C. moschata* with 12 events where chromosome/position/HMA copy number are conserved. Conversely, although the *Cucumis* genera shows nine events, HMA genes are not positionally conserved.

In addition, gene duplication events of the HMA gene family were found in all species, the *Cucurbita* genera being the highest. Interestingly, tandem duplication occurs in pairs labeled as *CamHMA2-3*, *CamHMA4-5*, *CcoHMA2-3*, *ClaHMA3-4*, *CmuHMA3-4*, *CmeHMA9-10*, *CmaHMA1-2*, *CmaHMA7-8*, *CmoHMA1-2*, *CmoHMA7-8*, *CpeHMA4-5*, and *CpeHMA9-10* while in *C. sativus* and *L. siceraria*, no tandem duplications were found. The non-synonymous rate (Ka), the synonymous rate (Ks), the Ka/Ks, and the duplication date using the Ks values of these pairs are presented in Table 2. In general, the Ka/Ks of tandem and segmental duplicates is less than 1, indicating they were under purifying selection. Moreover, the Ks of *Citrullus* duplication pairs ranges from 0.27 to 0.47 with divergent times that range from 21 to 36 MYA; similarly, in *Cucumis* and *L*. *siceraria*, the Ks value ranges from 0.27 to 0.44 and 0.33 to 0.44, and the divergent times from 20 to 33 MYA and 24 to 34 MYA, respectively. Nevertheless, *Cucurbita* duplication pairs’ Ks values range from 0.01 to 0.53 and divergent times from 1.27 to 40.88, which means a spectrum range of both, synonymous mutation, as well as diversification time. Interestingly, the tandem duplicates *CmaHMA7-8*, *CmoHMA7-8*, and *CpeHMA10-CpeHMA9* were speculated to diverge in recent time, <3 MYA ago, in contrast to the *Citrullus*, *Cucumis*, and *L. siceraria* duplicates that resulted >20 Mya.

### 3.4. Conserved Motif Analysis and Cis-Elements of HMA Proteins

**Figure 5:**
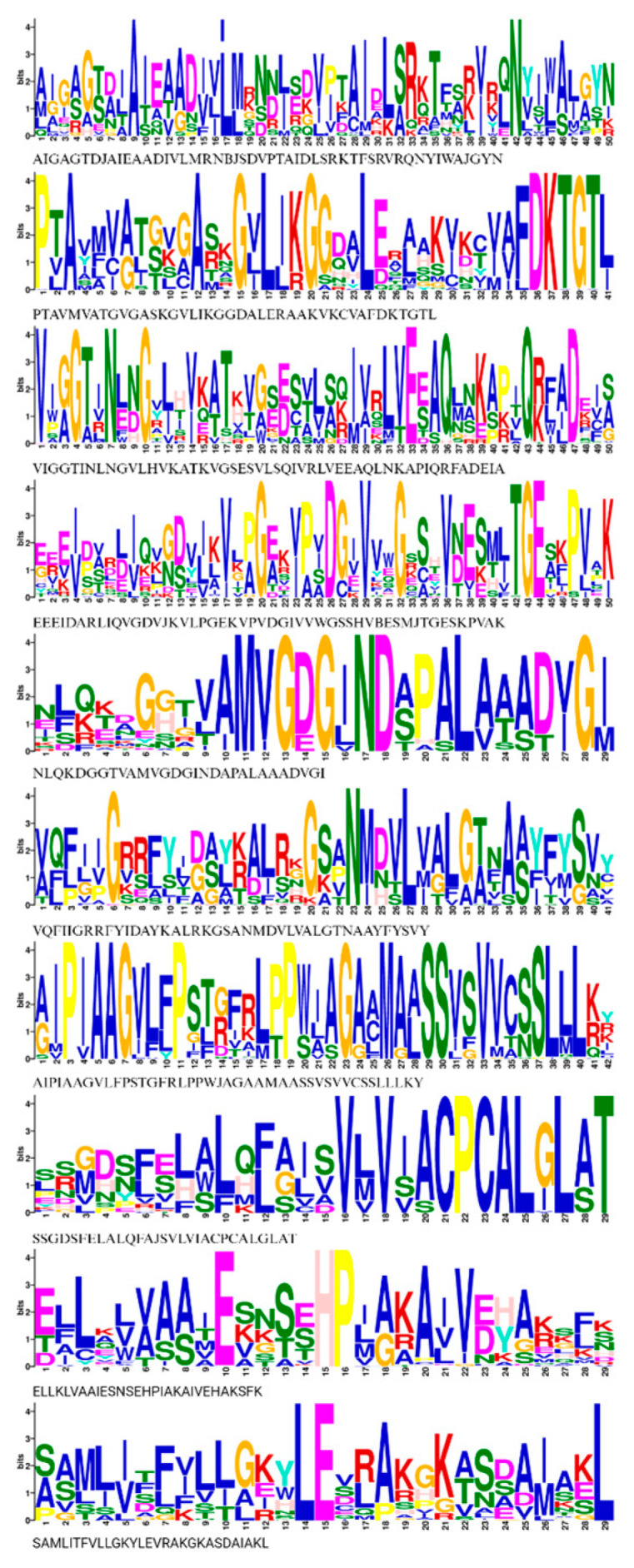
Common conserved motifs of HMA proteins among Cucurbitaceae plants: *C. amarus*, *C. colocynthis*, *C. lanatus*, *C. mucusospermus*, *C. melo*, *C. sativus*, *C. maxima*, *C. moschata*, *C. pepo*, and *L. siceraria*. The overall height of the stack indicates the degree of sequence conservation. The height of residues suggests the relative frequency of each residue at that position. Typed sequences of motifs are represented below each stack.

We identified ten common conserved motifs in HMA proteins by analyzing motif composition with MEME motif analysis, as shown in Figure 5. Common motifs ranged in length from 29 to 50 aa. The position and number of motifs vary according to the substrate specificity of Cu/Ag and Zn/Cd/Co/Pb clades (Figure S1). Furthermore, a ScanProsite analysis indicated that most of the motifs in the Cucurbit HMA family were associated with common functions of ATPase, such as the E1–E2 ATPase phosphorylation site, which is integral to the ATPase function. Details of the ten conserved motifs in common and for each Cucurbit species are given in Table S3.

To identify common putative *cis*-elements that can influence the expression of Cucurbit HMA genes, 2000-bp DNA sequences upstream of the start codon (ATG) for the HMA proteins for each species were analyzed using the PlantCARE database. There were 20 common elements identified associated with environmental stresses and plant hormonal processes. Table S4 shows all the identified common regulatory elements for the Cucurbit HMA family. The *cis*-regulatory elements associated with stress responses and possibly involved in the activation against heavy metals were TC-rich repeats, engaged in defense and stress response, LTR, associated with low temperatures, TCA cis-elements, related to salicylic acid that has an attenuation against biotic and abiotic stresses, and associated with heavy metal toxicity. 

### 3.5. GO Annotation of HMA Proteins

**Figure 6:**
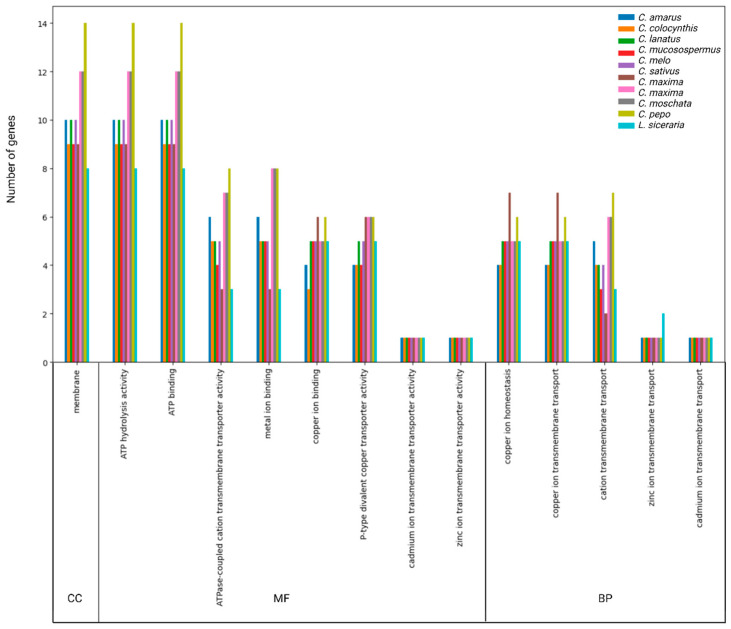
Gene ontology analysis results for Cucurbit species. Cellular Component (CC), Molecular Function (MF), and Biological Processes (BP) were identified with the OmicsBox program. Specific results for genes of each species are found in Supplementary Table S5.

The GO analysis performed with Omics Box suggested the putative participation of HMA genes in multiple biological processes, molecular functions, and cellular components (Figure 6 and Table S5). For instance, all 103 HMA proteins identified were predicted to be found in the membrane as a cellular component. Likewise, all HMA proteins were associated with the ATP-hydrolysis and binding activity for molecular function, followed by ATPase-coupled cation transmembrane transport activity. HMA proteins were involved in copper, cadmium, zinc transport, homeostasis, and metal ion binding for biological processes.

### 3.6. Gene Expression Pattern of HMA Genes in Tissues of Cucurbita Pepo under Cu and as Treatments

**Figure 7:**
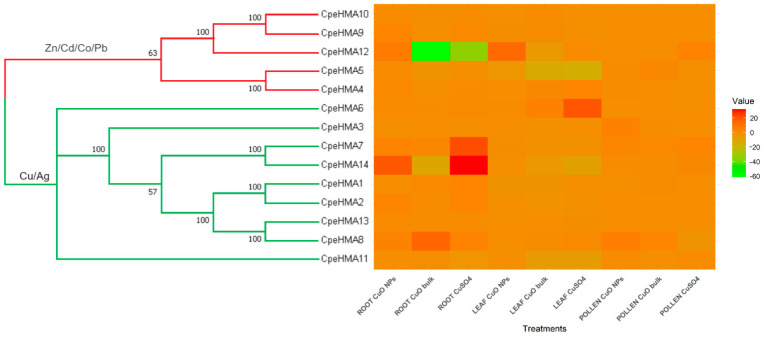
Phylogenetic tree and heat map of gene expression levels of HMA genes in root, leaf, and pollen tissues of *C. pepo* exposed to CuO NPs, bulk CuO, and bulk CuSO_4_ (Accession: GSE173716). The bar to the right of the heat map represents normalized expression values in each treatment.

To gain insights into the expression patterns and functions of HMA genes in *C. pepo*, we analyzed the expression profiles of CpeHMA genes. We based our analysis on their FPKM values derived from RNA-seq data acquired during copper (Cu) treatments, a heavy metal known to be linked with the HMA gene family. This initial investigation was a foundation for comparing subsequent expression results under arsenic exposure and validating our assumptions regarding gene functionality (Table S6). A phylogenetically clustered heatmap was used to visualize each gene’s specific gene expression patterns in root, leaf, and pollen tissue under CuO nanoparticles, bulk CuO, and bulk CuSO_4_ (Figure 7). Genes in clade Cu/Ag exhibited high expression levels, whereas genes in clade Zn/Cd/Co/Cu exhibited low expression levels. *CpeHMA14*, a member of the Cu/Ag clade, exhibited the highest expression among treatments and tissues, whereas *CpeHMA12*, a member of the Zn/Cd/Co/Pb clade, exhibited the lowest expression. The maximum fold-change expression levels of CpeHMA genes were detected in root and leaf tissues, while low or relatively no expression was found in pollen tissues. Members of the Cu/Ag clade, *CpeHMA7* and *CpeHMA8*, were highly expressed under bulk CuO and CuSO_4_ with a 21-fold and 14-fold change, respectively. On the other hand, the highest expressed gene for leaf tissue was also a member of the Cu/Ag family, *CpeHMA6*, with 19- and 5-fold alterations under bulk CuSO_4_ and CuO, respectively. *CpeHMA3* and *CpeHMA8*, members of the Cu/Ag clade, had the maximum expression levels in pollen under CuO NPs, with a five- and six-fold change, respectively.

**Figure 8:**
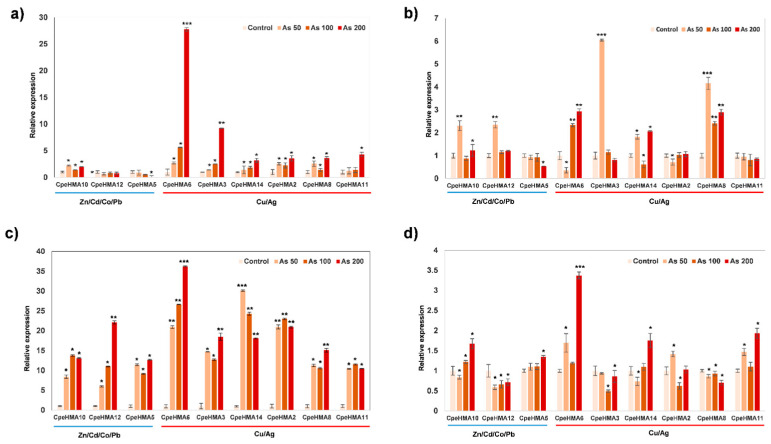
Expression pattern of HMA genes from *C. pepo* in different tissues under As stress. Relative expression levels of the CpHMA genes in (**a**) root, (**b**) leaf, (**c**) flower, and (**d**) fruit tissue of *C. pepo* plants treated with 50, 100, and 200 μM of arsenic in soil were determined with qRT-PCR. The gene expression level for each HMA gene in the control plants with no As was normalized to 1, as the 2^^ΔΔCT^ method suggests. The results represent the means of the biological replicates with their standard deviation represented as error bars. “*”, “**”, and “***” indicate genes statistically significantly differentially expressed between the treatment and the control using a *t*-test at the level of *p* ≤ 0.05, *p* ≤ 0.01, and *p* ≤ 0.001, respectively.

Based on gene homology and duplication events, we evaluated the expression pattern of nine *C. pepo* HMA genes (*CpeHMA2*, *CpeHMA3*, *CpeHMA5*, *CpeHMA6*, *CpeHMA8*, *CpeHMA10*, *CpeHMA11*, *CpeHMA12*, and *CpeHMA14*) in root, leaf, flower, and fruit tissues under 50 µM, 100 µM, and 200 µM As treatment through RT-qPCR. Notably, no downregulated genes were observed among all the evaluated HMA genes and tissues. However, genes belonging to the Cu/Ag clade exhibited significantly higher differential expression across all tissues (Figure 8). Interestingly, *CpeHMA6* showed upregulation in all As treatments across all tissue types. *CpeHMA3* displayed the highest expression in leaf tissue with a six-fold upregulation under the 50 µM treatment. In roots, *CpeHMA6*, *CpeHMA3*, *CpeHMA11*, *CpeHMA2,* and *CpeHMA8* exhibited differential expression with fold changes of 27, 9, 4, and 3, respectively, in the 200 µM treatment. Moreover, the flower tissue exhibited the most remarkable fold-change pattern, as all the studied genes showed upregulation across all treatments. *CpeHMA6* consistently exhibited significant expression with a 36-fold change under the 200 µM treatment, while *CpeHMA2* and *CpeHMA14* showed fold changes of >20 and >30, respectively, among different treatments. In contrast, the expression of the studied CpeHMA genes did not show significant differential expression in leaf and fruit tissues compared to root and flower tissues. The exception was *CpeHMA6*, which exhibited a notable three-fold upregulation under the 200 µM treatment in both leaf and fruit tissues.

## 4. Discussion

The HMA “heavy metal ATPase transporter” is a type of ATPase known as the P_1B_-type ATPase. It belongs to the P-type ATPase family, comprising ion pumps utilizing energy from ATP hydrolysis to uptake, translocate, compartmentalize, and detoxify heavy metal ions within plant cells [9,16]. Although HMA members have been identified and analyzed in *Arabidopsis* [36] and several crops such as rice [18], soybeans [20], and *Populus* [19], a comprehensive identification and characterization of this gene family in the Cucurbits have not been performed. In this study, a total of 103 heavy metal ATPase (HMA) proteins were identified across ten different Cucurbit species. The subsequent phylogenetic analysis of the HMA gene family revealed the division of HMA proteins into two distinct subfamilies (Zn/Co/Cd/Pb P1B-ATPase and the Cu/Ag P1B-ATPase) based on their structural and functional characteristics as described in previous studies [15,20,28]. *Cucurbita* plants, especially *C. pepo* with 14 members, exhibited a higher abundance of HMA proteins, despite having a smaller genome size (271.4 Mb for *C. maxima*, 269.9 Mb for *C. moschata*, and 263 Mb for *C. pepo*) [47,48] in comparison to Cucumis (375 Mb for *C. melo*) [49] and *Citrullus* (425 Mb for *C. lanatus*) [50], indicating that genome size may not have a positive correlation with the number of HMA family members. The length of sequences and isoelectric points of proteins significantly varied, indicating a high degree of diversification among the HMA genes in Cucurbits. Moreover, the domain structure of HMA genes from the significant clades was similar to the pattern shown in *Arabidopsis* [15]. Nevertheless, it is important to mention that all HMA Cucurbit genes possess a hydrolase domain since several HMA genes in other species have a lack or disruption of this domain, such as *MtHMA8* in Medicago and *ZmHMA9* in *Zea mays* [21,28]. This suggests that all Cucurbit HMA proteins may play an active role in metal transport due to ATP-hydrolysis-dependent mechanisms of energy required for transport [8,9]. Additionally, protein localization is the fundamental concept for understanding interactions at the systems’ level, and the function of transporters is inextricably linked to their subcellular localization [51]. In *Arabidopsis*, *AtHMA2* is expressed mainly in vascular tissues [52]; however, previous studies in both *Arabidopsis* and *Oryza sativa* have demonstrated that different HMAs exhibit diverse subcellular localizations [19,53]. In Cucurbits, all HMA proteins were predicted to localize within the cell membrane. Membrane proteins play a vital role in regulating plant responses to heavy metal stress, as they facilitate the transport of metals across membranes, thereby contributing to metal homeostasis and detoxification processes [54]. Expression of *CsHMA3* and *CsHMA4* was found to confer tolerance to Cd and Zn with metal efflux tolerance and accumulation of Cd and Pb through sequestration, proving to be part of the Zn/Co/Cd/Pb clade and suggesting its role in plant translocation from the plasma membrane and bioaccumulation of these metals into the vacuoles [34]. However, experimental validation is needed to locate HMAs and understand their role in other Cucurbits.

Furthermore, an important feature of P_1B_-ATPases is the presence of soluble metal binding domains (MBDs) that regulate transport activity [55]. The conserved structure characteristics of two cysteines (CxxC) of the HMA domain give HMA genes the basic function of binding metal ions through thiol groups [56,57]. P_1B_-type ATPases are capable of driving the efflux out of cells of both essential transition metal ions (e.g., Zn^2+^, Cu^+^, and Co^2+^) and toxic metal ions (e.g., Ag^+^, Cd^2+^, and Pb^2+^), contributing to their homeostasis maintenance [15,32]. Previous studies on members of the HMA gene family in *Arabidopsis* focused on heavy metal stress. Several genes, i.e., *AtHMA4*, *AtHMA2*, and *AtHMA3*, have been identified as Cd transporters involved in transporting Cd across the cell membrane and from the cytoplasm to the vacuole [10,11]. However, the molecular basis of HMA metal ion specificity remains unclear [58]. According to Smith et al. [8], HMA proteins appear to have functional roles in transporting manganese, iron, nickel, and other thiophilic heavy metals and metalloids such as arsenic. In plants, arsenic can easily enter through phosphate (P) transporters (arsenate) and aquaporin channels (arsenite), inhibiting plant growth and reducing crop yield [59,60,61]. After entering the plant, arsenic can be sequestered in the form of As-cysteine-rich peptides such as phytochelatins and then translocated into vacuoles mainly by ABC transporter subfamily C (ABCC) [62,63,64]. Nonetheless, different studies have reported alternative and independent arsenic transporters, such as the silicon transporters *Ls1* and *Ls2* that transport As (III) and the peptide transporter *OsPTR7* associated with the translocation of methylated-As species in *Oryza sativa* [24,59,65,66].

Although HMA proteins have not been previously characterized in arsenic transport, transcriptomic analyses showed that P-type ATPase genes were upregulated in roots and shoots of the hyper-accumulator *Pteris vitatta* in response to arsenic, which implies the role of P-type ATPase in the translocation of this metalloid [35]. Additionally, vacuolar proteomics showed that P-type ATPases were highly abundant compared to other metal transporters under arsenic stress. Likewise, previous studies have also reported the participation of non-elucidated transporters for arsenic in *Pteris vitatta* when treated with a mix of Ag-As due to the inhibition of the entrance, translocation, and the enhancement of As tolerance when Ag and As are supplemented simultaneously; it is known that Ag transport and homeostasis across plant tissues are mediated via HMA proteins from the Cu/Ag clade [67,68]. However, metal homeostasis in plants must be regulated using several complex processes [54], and the collaboration of transporters in different tissues may play an important role in plant metal distribution [19].

In order to understand the role of HMAs, we analyzed the gene expression levels from *C. pepo* genes (*CpeHMA*) in roots, leaves, and pollen from *C. pepo* under Cu treatments [42], a well-recognized HMA-related metal from proteins in the Ag/Cu clade, and arsenic stress in roots, leaves, flowers, and fruit. Under either Cu or As, genes that belong to the Zn/Co/Cd/Pb clade, *CpeHMA2*, *CpeHMA4*, *CpeHMA5*, CpeHMA9, *CpeHMA10, CpeHMA11*, and *CpeHMA12*, exhibited low to no expression levels in all tissues, whereas genes in the Cu/Ag clade such as *CpeHMA3*, *CpeHMA6*, *CpeHMA7*, *CpeHMA8*, and *CpeHMA14* in the Cu treatments and *CpeHMA3*, *CpeHMA6*, *CpeHMA8*, and *CpeHMA14* in the As treatments were highly expressed. *CpeHMA6* showed a high upregulation in leaf tissue under Cu treatment and in root and flower tissues under As stress. It was observed from the phylogenetic tree that *CpHMA6* is orthologous to *AtHMA8* from *Arabidopsis*, which is related to the Cu transport through the thylakoid membrane [69,70,71]. Cu is an essential metal due to its function as an enzyme cofactor for a number of physiological processes [72]. Nevertheless, As (V) can act as a P analog in the phosphorylation process that occurs in the thylakoid membrane, leading to the disruption of the ATP production process and thus threatening the energy homeostasis of the cell [59,73].

Moreover, the *CpeHMA3* gene, which is classified within the Cu/Ag clade, exhibited significant upregulation in response to both Cu and As treatments. Notably, the Cu/Ag clade lacks annotated HMA domains, a distinctive feature of this particular clade. The absence of highly conserved regions, particularly those associated with similar functionality observed in other species, can have an impact on both the affinity of the protein for various ionic metals and its inherent characteristics, such as heavy metal binding properties [13]. Moreover, *CpeHMA8* is an orthologue of *AtHMA5* and *OsHMA5* in *Arabidopsis* and *Oryza sativs*, respectively, while *CpeHMA14* is an orthologue of *AtHMA7*. *AtHMA5* is located in the plasma membrane and is involved in the Cu translocation from roots to shoots or Cu detoxification of roots [74]. *OsHMA5* is involved in loading Cu to the xylem of the roots and other organs [33]. Nevertheless, a study in *Populus trichocarpa* suggested that *PtHMA5* may differ in function from *AtHMA5* and *OsHMA5*, where it was found to have a significant role in Ag detoxification in addition to Cu detoxification [19]. Highly expressed *CpeHMA7* in root tissues under CuSO_4_ is orthologous to *AtHMA7*, also known as *RAN1*. The *AtHMA7* gene has been recognized as an ATP-dependent copper transporter that interacts with the ethylene receptor *ETR1*, which is primarily found in the endoplasmic reticulum regulating plant growth and development [75]. Therefore, considering the high expression observed in *CpeHMA3*, *CpeHMA6*, *CpeHMA7*, *CpeHMA8,* and *CpeHMA14* across the different tissues under Cu and As stress, it is plausible to hypothesize that HMA proteins may play a role in the transportation and tolerance mechanisms of arsenic in *Cucurbita pepo,* similar to their involvement in copper transport. However, further study and confirmation are required to elucidate the specific molecular pathways with which these CpeHMA genes respond to arsenic stress.

Moreover, through the examination of cis-regulatory elements in the HMA gene family, it has been observed that all genes harbor multiple cis-elements associated with abiotic stress, including those related to heavy metals [27,76]. Other *cis*-elements identified were the ABRE, ARE elements, LTR, and TC-rich repeats, which are associated with various stress responses, such as abscisic acid stress, anaerobic induction, low-temperature stress, defense mechanisms, and oxidative stress, which may be involved in generating a response to arsenic-induced oxidative stress [26,28]. Consequently, these findings suggest that the HMA genes in Cucurbits may be activated and potentially play a role in responding to other stress conditions.

## 5. Conclusions

Metal transporters play vital roles in distributing and transforming essential, non-essential, and even toxic metal ions in plants. This study comprehensively analyzed the HMA gene family in ten Cucurbit species. A total of 103 HMA genes from species of the Cucurbitaceous family were characterized and classified into two groups based on a phylogenetic analysis and their structural characteristics. According to their evolutionary metal association, the Cucurbit HMA genes had conserved or divergent gene structures, protein motif patterns, and cis-regulatory elements. The expression profiles of *CpeHMA* genes in various tissues/organs of *C. pepo* in response to both Cu and As stress indicate that the members of this gene family might be involved in transporting As metal ions across Cucurbit tissues, especially *CpeHMA6*. This information is valuable for functional investigation and understanding alternative molecular mechanisms responding to As stress in Cucurbits and other crops.

## Figures and Tables

**Table 1:** Comparative analysis of HMA proteins between Cucurbitaceae and other plant species and their distribution in the Zn/Cd/Co/Pb and Cu/Ag major clades.

Species	HMA Clade		Total
	Zn/Cd/Co/Pb	Cu/Ag	
*Arabidopsis thaliana*	4	4	8
*Brassica napus*	17	14	31
*Citrullus amarus*	4	6	10
*Citrullus colocynthis*	4	5	9
*Citrullus lanatus*	4	6	10
*Citrullus mucusospermus*	4	5	9
*Cucumis melo*	4	6	10
*Cucumis sativus*	3	6	9
*Cucurbita maxima*	5	7	12
*Cucurbita moschata*	5	7	12
*Cucurbita pepo*	5	9	14
*Glycine max*	6	14	20
*Hordeum vulgare*	3	6	9
*Legenaria siceraria*	3	5	8
*Linum usitatissimum*	4	8	12
*Medicago truncatula*	2	7	9
*Morus alba*	2	6	8
*Oryza sativa*	3	6	9
*Populus trichocarpa*	2	10	12
*Pyrus bretschneideri*	1	7	8
*Sorghum bicolor*	4	7	11
*Fagopyrum tataricum*	2	5	7
*Zea mays*	5	6	11

**Table 2:** Ka, Ks, Ka/Ks ratio, and divergent time of the duplicated HMA genes in Cucurbitaceous plants.

Species	Pair	Gene Names	Ka	Ks	Ka/Ks Ratio	Duplication Type	MYA ^1^
*C. amarus*	1	*CamHMA2-CamHMA3*	0.5081	0.4684	1.0846	Tandem	35.70
	2	*CamHMA1-CamHMA6*	0.1036	0.4185	0.2474	Segmental	31.90
	3	*CamHMA8-CamHMA10*	0.1208	0.3328	0.3629	Segmental	25.36
	4	*CamHMA7-CamHMA9*	0.2087	0.4635	0.4503	Segmental	35.33
	5	*CamHMA4-CamHMA5*	0.0796	0.2789	0.2855	Tandem	21.25
*C. colocynthis*	1	*CcoHMA1-CcoHMA4*	0.1056	0.4402	0.2400	Segmental	33.55
	2	*CcoHMA7-CcoHMA9*	0.1192	0.3347	0.3562	Segmental	25.51
	3	*CcoHMA6-CcoHMA8*	0.2019	0.4616	0.4374	Segmental	35.18
	4	*CcoHMA2-CcoHMA3*	0.0823	0.2832	0.2907	Tandem	21.59
*C. lanatus*	1	*ClaHMA2-ClaHMA8*	0.3042	0.4742	0.6415	Segmental	36.14
	2	*ClaHMA1-ClaHMA6*	0.1059	0.4301	0.2463	Segmental	32.78
	3	*ClaHMA5-ClaHMA10*	0.1174	0.3248	0.3615	Segmental	24.75
	4	*ClaHMA7-ClaHMA9*	0.2128	0.4653	0.4573	Segmental	35.46
	5	*ClaHMA3-ClaHMA4*	0.0832	0.2848	0.2922	Tandem	21.70
*C. mucusospermus*	1	*CmuHMA1-CmuHMA5*	0.1075	0.4420	0.2431	Segmental	33.69
	2	*CmuHMA7-CmuHMA9*	0.1212	0.3426	0.3538	Segmental	26.11
	3	*CmuHMA6-CmuHMA8*	0.2076	0.4521	0.4591	Segmental	34.46
	4	*CmuHMA3-CmuHMA4*	0.0847	0.2817	0.3009	Tandem	21.47
*C. melo*	1	*CmeHMA6-CmeHMA7*	0.1115	0.4288	0.2599	Segmental	32.68
	2	*CmeHMA2-CmeHMA4*	0.2848	0.3743	0.7608	Segmental	28.52
	3	*CmeHMA9-CmeHMA10*	0.0771	0.2719	0.2835	Tandem	20.72
	4	*CmeHMA3-CmeHMA5*	0.2542	0.3687	0.6894	Segmental	28.10
*C. sativus*	1	*CsaHMA6-CsaHMA8*	0.1077	0.4426	0.2434	Segmental	33.73
	2	*CsaHMA1-CsaHMA2*	0.1225	0.3678	0.3330	Segmental	28.03
	3	*CsaHMA5-CsaHMA7*	0.2109	0.4253	0.4958	Segmental	32.41
	4	*CsaHMA4-CsaHMA9*	0.3201	0.4293	0.7455	Segmental	32.72
*C. maxima*	1	*CmaHMA7-CmaHMA8*	0.0256	0.0308	0.8303	Tandem	2.35
	2	*CmaHMA6-CmaHMA9*	0.0272	0.1394	0.1951	Segmental	10.62
	3	*CmaHMA1-CmaHMA2*	0.0703	0.2765	0.2543	Tandem	21.07
	4	*CmaHMA3-CmaHMA11*	0.2830	0.4255	0.6651	Segmental	32.43
	5	*CmaHMA5-CmaHMA10*	0.0186	0.1056	0.1762	Segmental	8.05
*C. moschata*	1	*CmoHMA3-CmoHMA11*	0.3381	0.5363	0.6304	Segmental	40.88
	2	*CmoHMA1-CmoHMA2*	0.0874	0.3327	0.2628	Tandem	25.36
	3	*CmoHMA5-CmoHMA10*	0.0190	0.1179	0.1612	Segmental	8.99
	4	*CmoHMA6-CmoHMA9*	0.0241	0.1136	0.2125	Segmental	8.66
	5	*CmoHMA7-CmoHMA8*	0.0130	0.0167	0.7802	Tandem	1.27
*C. pepo*	1	*CpeHMA4-CpeHMA5*	0.0981	0.3070	0.3196	Tandem	23.39
	2	*CpeHMA7-CpeHMA14*	0.0294	0.1337	0.2198	Segmental	10.19
	3	*CpeHMA6-CpeHMA11*	0.2511	0.4202	0.5975	Segmental	32.03
	4	*CpeHMA9-CpeHMA10*	0.0170	0.0301	0.5649	Tandem	2.29
	5	*CpeHMA8-CpeHMA13*	0.0183	0.1176	0.1562	Segmental	8.96
*L. siceraria*	1	*LsiHMA5-LsiHMA8*	0.1290	0.3793	0.3400	Segmental	28.91
	2	*LsiHMA3-LsiHMA4*	0.2196	0.4498	0.4881	Segmental	34.28
	3	*LsiHMA1-LsiHMA7*	0.2933	0.3786	0.7748	Segmental	28.85
	4	*LsiHMA2-LsiHMA6*	0.0856	0.3274	0.2615	Segmental	24.95

^1^ million years ago.

## Data Availability

The data presented in this study are available in the article Supplementary Material.

## Supplementary Materials

The following supporting information can be downloaded at: https://www.mdpi.com/article/10.3390/genes14101877/s1, Figure S1: Position of the ten common motifs in protein sequences of Cucurbits. Sequence name is shown on the left side of each sequence, and the legend of each motif with the typed sequence is presented below all sequences; Table S1: Primer sequences of selected CpeHMA genes evaluated with RTqPCR under Arsenic stress; Table S2: Basic information on the HMA gene family in ten Cucurbit species; Table S3: Common Conserved Motifs Present in HMA Family in Cucurbit species; Table S4. Common putative cis-elements identified in the promoter sequences of HMA protein genes in Cucurbit species. Table S5: Gene ontology annotation results of HMA genes in Cucurbit species; Table S6: Normalized RNA-seq data from *C. pepo* under Cu stress.
