# Supplementary material for: A Genome-Wide Identification and Comparative Analysis of the Heavy-Metal-Associated Gene Family in Cucurbitaceae Species and Their Role in Cucurbita pepo under Arsenic Stress
Source: Genes (Basel). 2023 Sep 27;14(10):1877. doi: 10.3390/genes14101877 (PMC10606463; doi:10.3390/genes14101877)
Supplement: Supplementary file 1 [file genes-14-01877-s001.zip › Supplementary Figure.pdf]

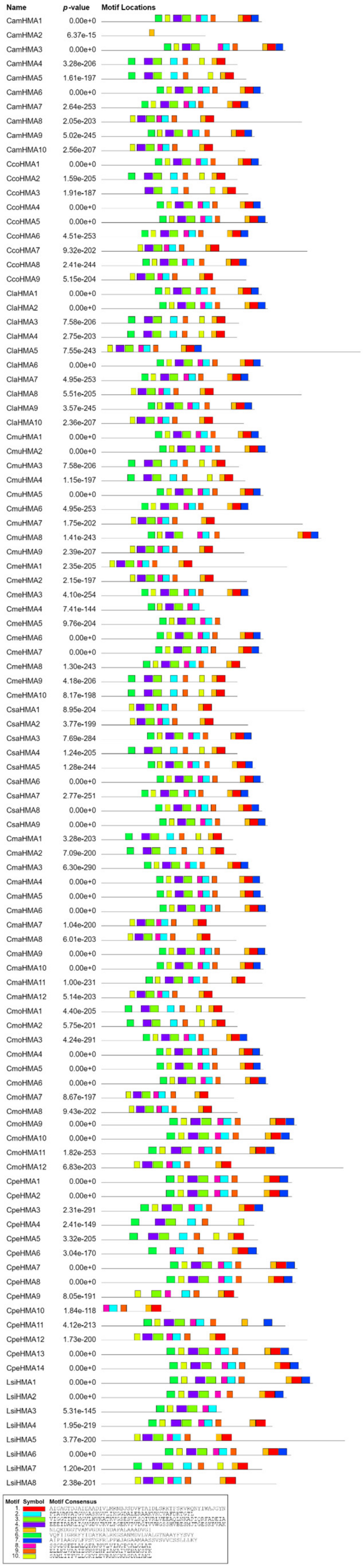

Name
p-value
Motif Locations
CamHMA1 0.00e+0
CamHMA2 6.37e-15
CamHMA3 0.00e+0
CamHMA4 3.28e-206
CamHMA5 1.61e-197
CamHMA6 0.00e+0
CamHMA7 2.64e-253
CamHMA8 2.05e-203
CamHMA9 5.02e-245
CamHMA10 2.56e-207
CcoHMA1 0.00e+0
CcoHMA2 1.59e-205
CcoHMA3 1.91e-187
CcoHMA4 0.00e+0
CcoHMA5 0.00e+0
CcoHMA6 4.51e-253
CcoHMA7 9.32e-202
CcoHMA8 2.41e-244
CcoHMA9 5.15e-204
ClaHMA1 0.00e+0
ClaHMA2 0.00e+0
ClaHMA3 7.58e-206
ClaHMA4 2.75e-203
ClaHMA5 7.55e-243
ClaHMA6 0.00e+0
ClaHMA7 4.95e-253
ClaHMA8 5.51e-205
ClaHMA9 3.57e-245
ClaHMA10 2.36e-207
CmuHMA1 0.00e+0
CmuHMA2 0.00e+0
CmuHMA3 7.58e-206
CmuHMA4 1.15e-197
CmuHMA5 0.00e+0
CmuHMA6 4.95e-253
CmuHMA7 1.75e-202
CmuHMA8 1.41e-243
CmuHMA9 2.39e-207
CmeHMA1 2.35e-205
CmeHMA2 2.15e-197
CmeHMA3 4.10e-254
CmeHMA4 7.41e-144
CmeHMA5 9.76e-204
CmeHMA6 0.00e+0
CmeHMA7 0.00e+0
CmeHMA8 1.30e-243
CmeHMA9 4.18e-206
CmeHMA10 8.17e-198
CsaHMA1 8.95e-204
CsaHMA2 3.77e-199
CsaHMA3 7.69e-284
CsaHMA4 1.24e-205
CsaHMA5 1.28e-244
CsaHMA6 0.00e+0
CsaHMA7 2.77e-251
CsaHMA8 0.00e+0
CsaHMA9 0.00e+0
CmaHMA1 3.28e-203
CmaHMA2 7.09e-200
CmaHMA3 6.30e-290
CmaHMA4 0.00e+0
CmaHMA5 0.00e+0
CmaHMA6 0.00e+0
CmaHMA7 1.04e-200
CmaHMA8 6.01e-203
CmaHMA9 0.00e+0
CmaHMA10 0.00e+0
CmaHMA11 1.00e-231
CmaHMA12 5.14e-203
CmoHMA1 4.40e-205
CmoHMA2 5.75e-201
CmoHMA3 4.24e-291
CmoHMA4 0.00e+0
CmoHMA5 0.00e+0
CmoHMA6 0.00e+0
CmoHMA7 8.67e-197
CmoHMA8 9.43e-202
CmoHMA9 0.00e+0
CmoHMA10 0.00e+0
CmoHMA11 1.82e-253
CmoHMA12 6.83e-203
CpeHMA1 0.00e+0
CpeHMA2 0.00e+0
CpeHMA3 2.31e-291
CpeHMA4 2.41e-149
CpeHMA5 3.32e-205
CpeHMA6 3.04e-170
CpeHMA7 0.00e+0
CpeHMA8 0.00e+0
CpeHMA9 8.05e-191
CpeHMA10 1.84e-118
CpeHMA11 4.12e-213
CpeHMA12 1.73e-200
CpeHMA13 0.00e+0
CpeHMA14 0.00e+0
LsiHMA1 0.00e+0
LsiHMA2 0.00e+0
LsiHMA3 5.31e-145
LsiHMA4 1.95e-219
LsiHMA5 3.77e-200
LsiHMA6 0.00e+0
LsiHMA7 1.20e-201
LsiHMA8 2.38e-201
Motif
Symbol
Motif Consensus
1.
2.
3.
4.
5.
6.
7.
8.
9.
10.

Figure S1. Position of the ten common motifs in protein sequences of Cucurbits. Sequence name is shown in the left side of each sequence and the legend of each motif with the typed sequence is presented below all sequences.
