# Supplementary figures and images for: A Genome-Wide Identification and Comparative Analysis of the Heavy-Metal-Associated Gene Family in Cucurbitaceae Species and Their Role in Cucurbita pepo under Arsenic Stress
Source: Genes (Basel). 2023 Sep 27;14(10):1877. doi: 10.3390/genes14101877 (PMC10606463; doi:10.3390/genes14101877)

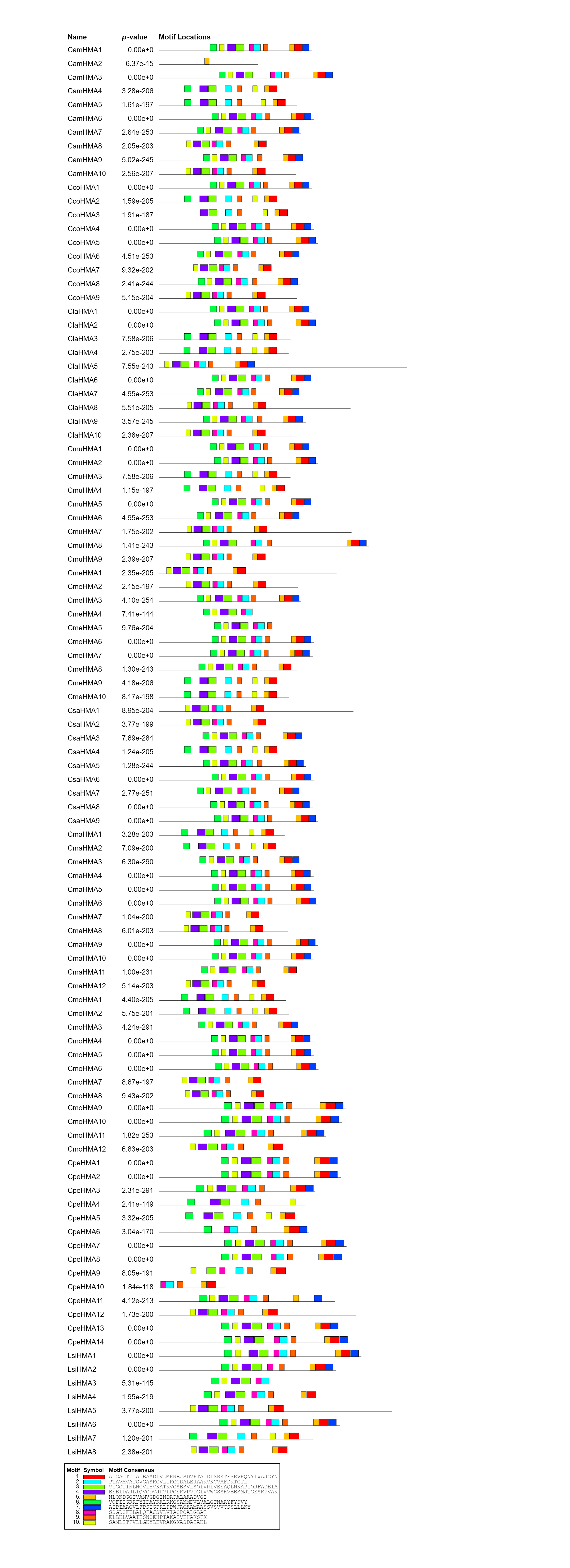

Supplement: Supplementary file 1 [file genes-14-01877-s001.zip › Supplementary Figure 1.png]
